# Metabolic tracing reveals novel adaptations to skeletal muscle cell energy production pathways in response to NAD ^+^ depletion

**DOI:** 10.12688/wellcomeopenres.14898.2

**Published:** 2019-09-17

**Authors:** Lucy A. Oakey, Rachel S. Fletcher, Yasir S. Elhassan, David M. Cartwright, Craig L. Doig, Antje Garten, Alpesh Thakker, Oliver D. K. Maddocks, Tong Zhang, Daniel A. Tennant, Christian Ludwig, Gareth G. Lavery

**Affiliations:** 1 Institute of Metabolism and Systems Research, University of Birmingham, UK, Birmingham, B15 2TT, UK; 2 Institute of Cancer Sciences, University of Glasgow, Glasgow, G61 1QH, UK

**Keywords:** NAD+, NAMPT, NR, metabolism, skeletal muscle, isotopic tracing, aspartate

## Abstract

**Background:** Skeletal muscle is central to whole body metabolic homeostasis, with age and disease impairing its ability to function appropriately to maintain health. Inadequate NAD ^+^ availability is proposed to contribute to pathophysiology by impairing metabolic energy pathway use. Despite the importance of NAD ^+^ as a vital redox cofactor in energy production pathways being well-established, the wider impact of disrupted NAD ^+^ homeostasis on these pathways is unknown.

**Methods:** We utilised skeletal muscle myotube models to induce NAD ^+^ depletion, repletion and excess and conducted metabolic tracing to provide comprehensive and detailed analysis of the consequences of altered NAD ^+^ metabolism on central carbon metabolic pathways. We used stable isotope tracers, [1,2-13C] D-glucose and [U- ^13^C] glutamine, and conducted combined 2D-1H,13C-heteronuclear single quantum coherence (HSQC) NMR spectroscopy and GC-MS analysis.

**Results:** NAD ^+^ excess driven by nicotinamide riboside (NR) supplementation within skeletal muscle cells resulted in enhanced nicotinamide clearance, but had no effect on energy homeostasis or central carbon metabolism. Nicotinamide phosphoribosyltransferase (NAMPT) inhibition induced NAD ^+^ depletion and resulted in equilibration of metabolites upstream of glyceraldehyde phosphate dehydrogenase (GAPDH). Aspartate production through glycolysis and TCA cycle activity was increased in response to low NAD ^+^, which was rapidly reversed with repletion of the NAD ^+^ pool using NR. NAD ^+^ depletion reversibly inhibits cytosolic GAPDH activity, but retains mitochondrial oxidative metabolism, suggesting differential effects of this treatment on sub-cellular pyridine pools. When supplemented, NR efficiently reversed these metabolic consequences. However, the functional relevance of increased aspartate levels after NAD ^+^ depletion remains unclear, and requires further investigation.

**Conclusions:** These data highlight the need to consider carbon metabolism and clearance pathways when investigating NAD ^+^ precursor usage in models of skeletal muscle physiology.

## Introduction

Nicotinamide adenine dinucleotide (NAD ^+^) is an essential cofactor responsible for facilitating oxidoreductase reactions throughout glycolysis, the tricarboxylic acid (TCA) cycle, and the electron transport chain (ETC) ^1, 2^. Maintaining NAD ^+^/reduced NAD ^+^ (NADH) redox homeostasis is important for effective ATP production, which is essential for metabolically active tissues to meet functional demand ^3^. NAD ^+^ has since also been recognised as a signalling molecule and is intracellularly consumed by enzymes such as sirtuins, poly-ADP-ribose polymerases (PARPs) and cADP-ribose synthases (CD38s). In order to maintain normal cellular function, it is critical for NAD ^+ ^to be replenished through either biosynthetic or salvage pathways ^4– 6^.

Pathological changes to skeletal muscle have consequences on whole body energy metabolism as seen in disease states such as obesity, diabetes and sarcopenia ^7– 9^. A reduction in NAD ^+^ levels of between 30 and 85% have been reported in the muscle tissue of aged mice with an associated impairment in mitochondrial function ^5, 10– 14^. Reduced NAD ^+^ availability may also be a molecular mechanism involved in the aetiology of muscle dysfunction in more common metabolic diseases, including obesity and type II diabetes ^10– 12^. Augmentation of muscle NAD ^+^ levels through precursor supplementation (i.e. nicotinamide riboside (NR), nicotinamide mononucleotide (NMN)) may be a means to improve metabolic capacity and function in a range of age-related disease states ^15^. NR and NMN are often supplemented in large doses and can lead to increased cellular NAD ^+^ content. Chronically increasing NAD ^+^ beyond normal physiological levels could have a wider impact on metabolic homeostasis, which is yet to be fully understood.

The role of NAD ^+^ in accepting electrons from glycolytic and TCA cycle metabolites enables oxidative phosphorylation and highlights the NAD ^+^/NADH redox couple as being vital to central carbon metabolism. Based on the decline in NAD ^+^ observed in chronic disease we studied the impact this may have on energy homeostasis. In light of the recent work showing NR can safely elevate NAD ^+^ concentration in human blood ^16^, alongside the increasing focus on NAD ^+^ precursors as a treatment strategy for metabolic diseases, we sought to explore the metabolic impact of altered NAD ^+^ levels in the context of skeletal muscle ^16– 18^.

Here we established scenarios of NAD ^+^ excess and depletion, previously modelled in other studies ^12, 19, 20^, and aimed to more intensively define the consequences of disrupted NAD ^+^ on essential metabolic pathways in skeletal muscle, providing advances to the current work in the field. We initially employed 1D- ^1^H-NMR, which has higher resolution and reproducibility than other quantification methods, such as GCMS ^21^. Further to this, we utilised metabolic tracing to understand the impact of altered NAD ^+^ levels on carbon metabolism. The stable isotope tracer [1,2- ^13^C] D-glucose was used to generate metabolite labelling patterns and visualised through advanced 2D- ^1^H, ^13^C-heteronuclear single quantum coherence (HSQC) nuclear magnetic resonance (NMR) spectroscopy and gas chromatography–mass spectrometry (GC-MS) analysis ^22^. This methodology allowed for in-depth analysis of glucose-derived carbon molecules, with the ability to determine information regarding specific metabolic pathways within different compartments ^23^. Detailed analysis of lactate and alanine enable greater understanding of the changes to glycolysis and the pentose phosphate pathway (PPP) within the cytosol. Investigation of glutamate and aspartate, using this method, can determine whether synthesis has occurred from pyruvate entering the mitochondria via pyruvate dehydrogenase or pyruvate carboxylase ^22, 23^. Further to this, information regarding the number of rounds of the TCA cycle can be determined, which once again can be used to understand changes to the activity of the TCA cycle in response to differing NAD ^+^ states.

Here we present detailed evidence of adaptations to pathway utilisation within central carbon metabolism in response to low NAD ^+^ levels in skeletal muscle, which are reversible with short-term NR supplementation.

## Methods

Unless otherwise stated all materials and reagents were acquired from Sigma-Aldrich, UK

### Culture of the murine C2C12 muscle cell line

C2C12 myoblasts were grown in Dulbecco’s Modified Eagle’s Medium (DMEM) 25 mM glucose supplemented with 10% foetal bovine serum (FBS) and 1% Penicillin/Streptomycin (P/S). Once cells reached 70% confluence, differentiation medium was added (DMEM 25 mM glucose supplemented with 5% horse serum (HS) and 1% P/S) and the cells were cultured for 5 days with fresh medium added every other day, sufficient to differentiate myoblasts into mature contractile myotubes.

### Primary myotube culture

Experiments were conducted consistent with current UK Home Office regulations in accordance with the UK Animals (Scientific Procedures) Act 1986, and approved by the local Animal Welfare and Ethical Review Body. Gastrocnemius muscle was collected from 16-week-old male C57BL/6NJ mice and placed in 0.2% collagenase, to allow for myofibre detachment and digestion and then washed in DMEM. Myofibres were placed in wells coated with Matrigel (BD biosciences), with DMEM containing 30% FBS, 10% HS, 1% chick embryo extract (CEE), 1% P/S and 0.1% fibroblast growth factor (FGF). Following satellite cell migration the media was replaced with proliferation media; DMEM supplemented with 10% HS, 0.5% CEE and 1% P/S. Once cells had attained 70–80% confluence the media was replaced with differentiation media (DMEM supplemented with 2% HS, 0.5% CEE and 1% P/S) and left to differentiate for 6 days, as previously described ^24^.

### Cell treatments

Cells were either left as control or treated with 50 nM FK866 for 24 or 48 hours and then co-treated with 0.5 mM NR (Chromadex, California) for 4 hours. NMN treatments were the same as NR; 0.5 mM NMN for 4 hours. These concentrations were selected to provide a maximal effect on cellular NAD levels following previously published work in myotubes ^25^.

### ^13^C-glucose and ^13^C-glutamine labelling

Cells (2x10 ^6^) were seeded into 15 cm dishes and differentiated for 5 days. For glucose labelling, freshly made flux media (DMEM powder (without glucose, L-glutamine, phenol red, sodium pyruvate or sodium bicarbonate), diluted in 1 litre of distilled water, supplemented with 2 mM glutamine and 45 mM sodium bicarbonate) was added to the cells 24 hours prior to extraction; 10 mM ^13^C _2_-[1,2]-D-Glucose was then added and the cells were left to metabolise. For glutamine labelling the media was alternatively supplemented with 10 mM glucose, 45 mM sodium bicarbonate and 2 mM ^13^C _5_-glutamine.

### Extraction of metabolites from cells

Media was aspirated and plates were washed with ice-cold 0.9% saline solution before being placed on dry ice to quench metabolism prior to extraction of metabolites. 1.2 ml HPLC-grade methanol (-80°C) was added to the plate. The cells were scraped using a cell scraper and transferred to a falcon tube where 1.2 ml of HPLC-grade chloroform (-20°C) was added. The tube was rocked at 4°C for 10 minutes before 1.2 ml pre-chilled HPLC-grade H _2_O was added and left to rest on ice for 10 minutes. Separation of the polar and non-polar fractions was achieved through centrifugation at 4°C, 14,200 rpm for 15 minutes. Next, 2 ml of the polar fraction was stored for NMR analysis, with 200 µl removed for GC-MS. Both fractions were evaporated to dryness using a SpeedVac at 30°C for 4–5 hours and stored at -80°C until further analysis. Protein pellet weights were used for normalisation during analysis; extractions were conducted on at least three different experiments.

### NMR spectroscopy

Dried NMR samples were re-suspended in 100 mM sodium phosphate buffer containing 500 µM 4,4-dimethyl-4-silapentant-1-sulfonic acid (DSS) and 10% deuterium (D _2_O), pH 7.0. Samples were sonicated and transferred to glass vials before being moved to 1.7 mm NMR tubes using a Gilson robotic system.

A Bruker Avance III 600 MHz NMR spectrometer equipped with a 1.7 mm z-PFG TCI Cryoprobe was used to acquire 2D 1H,13C-HSQC NMR spectra. The HSQC spectra were acquired with echo/anti-echo gradient coherence selection with an additional pre-saturation for suppressing the water resonance. The spectral widths were 13.018 ppm and 160.0544 ppm in the direct and indirect dimension, 512 complex data points were acquired for the 1H dimension and 29.9927% (2457) out of 8192 complex data points were acquired for the 13C indirect dimension using a non-uniform sampling scheme. The interscan relaxation delay was set to 1.5 s. 2D 1H,13C-HSQC spectra were reconstructed via the compressed sensing algorithm using the MDDNMR (version 2.5) and NMRPipe (version 9.2) software ^26– 28^. All spectra were processed without baseline correction as this can present challenges for the multiplet analysis procedure.

A total of 128 transients were acquired for each 1D- ^1^H NMR spectrum with a relaxation delay of 4 s. Each sample underwent automatic tuning and matching before they were shimmed (1D-TopShimm) to a DSS line width of <1 Hz prior to acquisition of the first spectrum. Total experiment time was 4 h 45 min per sample; ~15 min for 1D- ^1^H-NMR and 4.5 h for 2D- ^1^H, ^13^C-HSQC NMR spectra. All 1D-1H NMR spectra were processed using MetaboLab software (version 1). Data analysis was performed using MetaboLab with the methyl group of lactate used to calibrate the chemical shift ^29^. Prior to Fourier Transformation all 1D- ^1^H-NMR spectra were zero-filled to 131,072 data points, the chemical shift was then calibrated by referencing the DSS signal to 0 ppm and the spectra were manually phase-corrected. Once complete, the baseline correction was conducted using a spline function (ref: 61) and the spectra were exported into Bruker format to allow for metabolite identification and quantification using the Chenomx software package (ChenomxINC, version 7.0). All metabolite concentrations were normalised to pellet weight.

### GC-MS

Polar metabolites were solubilised in 2% methoxyamine hydrochloric acid (HCL) in pyridine. The samples were vortexed before being incubated at 60°C for 60 minutes. Following this incubation the derivatisation reagent was added; 60 μl N-tertbutyldimethylsilyl-N-methyltrifluoroacetamide (MTBSTFA) with 1% (w/v) tertbutyldimethyl-chlorosilane (TBDMSCI) (Sigma-Aldrich). The suspension was incubated for 1 h at 60°C in a closed tube to prevent evaporation. The samples were centrifuged at 13,000 rpm for 5 min and the clear supernatant was transferred to a chromatography vial with a glass insert (Thermo Fisher). Derivatised samples were analysed using an Agilent 7890B Series GC/MSD gas chromatograph with a medium polar range polydimethylsiloxane GC column (DB35-MS), in association with a mass spectrometer (GC-MS) (Agilent Technologies). Metabolite ion counts were normalised to pellet weight.

### Glycogen assay

Glycogen was extracted from differentiated C2C12s and quantified using a Glycogen Assay Kit (Sigma-Aldrich) according to the manufacturer’s instructions.

### Respirometry

C2C12s were differentiated for 5 days and treated with 50 nM FK866 for 48 or 72 h. Mitochondrial function was then determined using a two-chamber Oxygraph (OROBOROS Instruments) derived from polarographic oxygen flux measurements. Prior to loading into the chambers cells were removed from the plates by Trypsin and spun down to form a pellet before being suspended in 2 ml DMEM. Once within the chambers the cells were left to incubate for 10 min to measure their endogenous respiration. Following this 10 µg/2 ml of digitonin was added to permeabilise the cells. Assessment of oxidation capacity was carried out by sequentially subjecting the cells to differing concentrations of substrate as follows; 2 mM malate and 10 mM glutamate as a substrate for complex I and determination of complex I respiratory capacity, 5 mM increments of ADP until maximal oxidative phosphorylation and induction of state III respiration, 10 µM cytochrome c as a control for mitochondrial membrane damage, 20 mM succinate as a substrate for complex II and assessment of complex I + II respiratory capacity, 0.5 mM carbonyl cyanide- *4*-(trifluoromethoxy)phenylhydrazone (FCCP) titrations until maximal respiration increase to determine maximal uncoupled respiratory capacity, 0.5 µM rotenone for inhibition of complex I and assessment of complex II maximal respiratory capacity, and 2.5 µM antimycin A for inhibition of complex III to determine residual oxygen consumption. Following experiment chamber contents were collected, spun down, washed in PBS and a Bradford assay was conducted to calculate protein levels within each sample. These were then used to determine oxygen flux/mg of protein. Respiratory substrate stocks were diluted in ddH _2_O, while uncouplers and inhibitors were diluted in absolute ethanol.

### LCMS

Prepared samples were analysed on a LCMS platform consisting of an Accela 600 LC system and an Exactive mass spectrometer (Thermo Scientific). A Sequant ZIC-pHILIC column (4.6 mm × 150 mm, 5 μm) (Merck) was used to separate the metabolites with the mobile phase mixed by A= 20 mM ammonium carbonate in water and B= acetonitrile. A gradient program starting at 20% of A and linearly increasing to 80% at 30 min was used followed by washing (92% of A for 5 min) and re-equilibration (20% of A for 10 min) steps. The total run time of the method was 45 min. The LC stream was desolvated and ionised in the HESI probe. The Exactive mass spectrometer was operated in full scan mode over a mass range of 70–1,200 m/z at a resolution of 50,000 with polarity switching. The LCMS raw data was converted into mzML files by using ProteoWizard (v3.0.4472, 64-bit) and imported to MZMine (2.10) for peak extraction and sample alignment. A house-made database including all possible ^13^C isotopic m/z values of the relevant metabolites was used for the assignment of LCMS signals. Finally the peak areas were used for comparative quantification.

### Statistical analysis

All statistical analyses were carried out using the GraphPad Prism 6 software. One-way ANOVA analysis has been conducted on the concentrations of metabolites and percentages of label incorporation, with Dunnett’s multiple comparisons post hoc test. This was to test differences between the treatment groups with statistical significance shown by * p<0.05, ** p<0.01, *** p<0.001, compared to control.

## Results

### NAD ^+^ excess does not impact pathway use in central carbon metabolism

**Figure 1.**
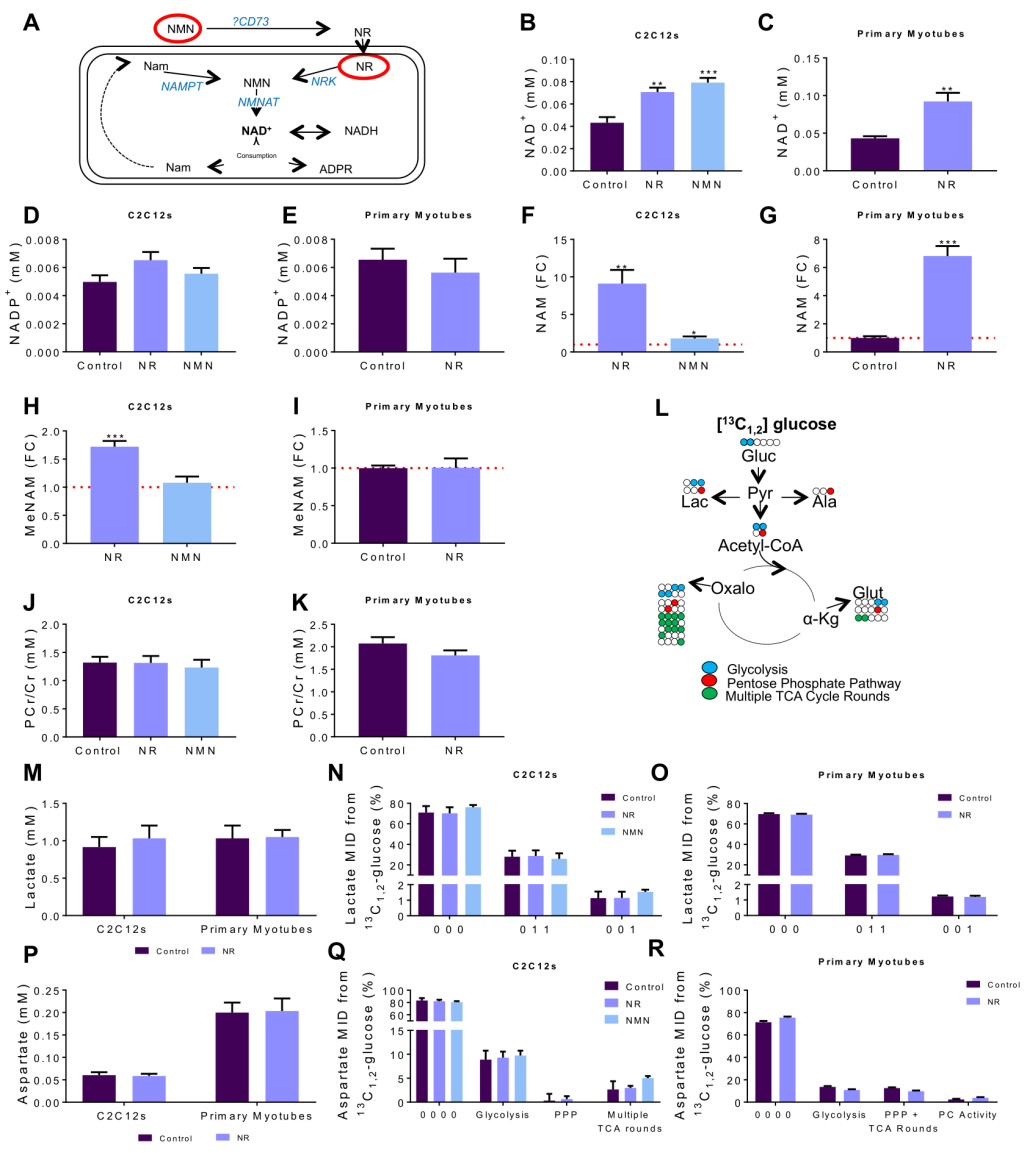
NAD ^+^ excess has no consequence on pathway use within central carbon metabolism. Differentiated C2C12 and primary myotubes were treated with NR or NMN (0.5 mM) for 4 hours, pathway shown in ( **A**). Key metabolites were identified and quantified using 1D-1H-NMR spectroscopy and used to inform on the energetic status of the cell; ( **B**, **C**) NAD ^+^, ( **D**, **E**) NADP ^+^. Fold-change compared to control were shown for ( **F**, **G**) nicotinamide (NAM) and ( **H**– **I**) methylated-nicotinamide (MeNAM); red line is representative of control levels. ( **J**, **K**) The phosphocreatine (PCr)/creatine (Cr) ratio. ( **L**) Resulting labelling patterns of glucose flux through metabolic pathways. Concentration and labelling patterns detected through combined analysis of 2D- ^1^H, ^13^C-NMR ( ^13^C _1,2_-glucose) and GC-MS data of ( **M**– **O**) lactate and ( **P**– **R**) aspartate. ^13^C represented by 1 with ^12^C represented by 0; % MID of total metabolite. All concentrations in mM. * p<0.05, ** p<0.01, ***p<0.005; One-way ANOVA performed on raw data; Dunnett’s multiple comparison test, treatment compared to control. All data are the mean ±SEM, C2C12s n=6. Primary myotubes n=4. FC, fold change compared to control; MID, mass isotopomer distribution.

NR and NMN supplementation as a strategy to increase or recover NAD ^+^ availability has been extensively used in models of pathophysiology associated with NAD ^+^ deficiency and impaired metabolism ^11^. The wider metabolic consequences of increasing cellular NAD ^+^ above physiological levels have yet to be established. Therefore, we first examined the consequences of NAD ^+^ excess on metabolic pathway use and energetic status in muscle cells. Using 1D- ^1^H-NMR spectroscopy we quantified changes to key metabolites involved in NAD ^+^ metabolism following 0.5 mM NR or NMN supplementation in control cells. As expected, 4-hour NR and NMN were effective at significantly elevating intracellular NAD ^+^ levels in C2C12 cells, with a similar significant increase seen in primary myotubes following NR supplementation ( Figure 1B, C). There was no effect of NR or NMN supplementation on NADP ^+^ in either C2C12s or primary myotubes ( Figure 1D, E). NAM, the breakdown product and precursor of NAD ^+^, was significantly elevated in cells treated with NR and NMN. NR had a greater effect on NAM with a 9-fold increase above control compared to NMN (1.8-fold) ( Figure 1F, G). NAM clearance was also elevated, with methylated NAM (MeNAM) levels significantly increased with NR but not NMN supplementation in C2C12s ( Figure 1H). Interestingly, there was no change in MeNAM after NR supplementation in primary myotubes despite a large increase in NAM ( Figure 1I). We used the phosphocreatine/creatine (PCr/Cr) ratio, to show there was no change in ATP demand as a result of NAD ^+ ^excess following supplementation with either precursor in C2C12s or primary myotubes ( Figure 1J, K).

To further characterise the effect of NAD ^+^ excess on carbon metabolism we used [1,2- ^13^C] D-glucose and conducted combined analysis of GC-MS and 2D- ^1^H, ^13^C-HSQC spectra to elucidate changes to metabolic pathway utilisation ( Figure 1L). The use of [1,2- ^13^C] D-glucose as an isotopic tracer delivers distinct labelling patterns based on whether the PPP has been utilised, which we may expect to change with varying NAD ^+^ levels. Lactate was used as a measure of glycolytic and PPP activity, with no change observed in concentration or pathway utilisation with NR or NMN ( Figure 1L–N). In order to identify TCA cycle changes aspartate was used as a surrogate; once again, no change in concentration or pathway use was observed ( Figure 1O–R). To investigate whether a longer supplementation of NR would induce changes to carbon metabolism we conducted a 24-hour NR study. Similar to the 4-hour supplementation period, both NAM and MeNAM levels were elevated suggesting increased clearance of NAD ^+^ metabolites ( Supplementary Figure 1C, D) but carbon metabolism was unchanged ( Supplementary Figure 1G–H).

These data show that both acute and chronic NAD ^+^ excess within a skeletal muscle cell results in increased NAM clearance but has no immediate effect on energy homeostasis or central carbon metabolism based on the endpoints measured. We proceeded to use the 4-hour NR supplementation for our NAD ^+^ rescue experiments to show the acute effects of NAD ^+^ repletion using NR.

### NR supplementation can rescue NAD ^+^ depletion and reverse FK866 induced energy stress

**Figure 2.**
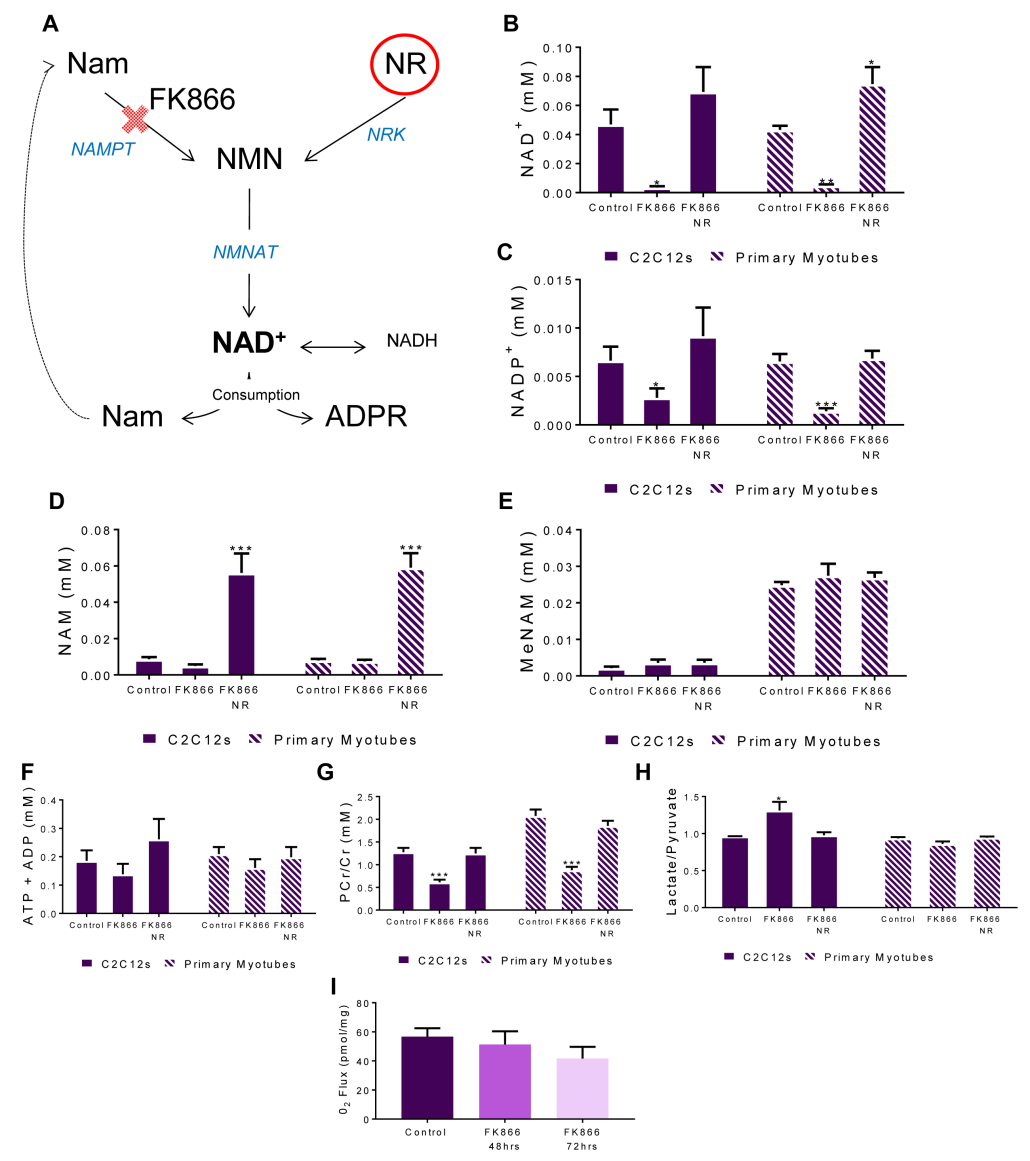
NAMPT inhibition induces energetic stress in skeletal muscle. Differentiated C2C12 and primary myotubes were treated with FK866 (50 nM) for 48 hours with and without NR (0.5 mM) for 4 hours, pathway shown in ( **A**). Key metabolites were identified and quantified using 1D- ^1^H-NMR spectroscopy and used to inform on the energetic status of the cell; ( **B**) NAD ^+^, ( **C**) NADP ^+^, ( **D**) nicotinamide (NAM), ( **E**) methylated-nicotinamide (MeNAM), ( **F**) combined ATP and ADP and ( **G**) the phosphocreatine (PCr)/ creatine (Cr) ratio. ( **H**) Lactate/Pyruavte ratio from GC-MS analysis using the m=2 portion of the MID. ( **I**) High resolution respirometry was carried out on differentiated C2C12s treated with 50 nM FK866 for 24, 48 or 72 hours. * p<0.05, ** p<0.01, ***p<0.005; One-way ANOVA performed on raw data; Dunnett’s multiple comparison test, treatment compared to control. All data are the mean ±SEM, C2C12s n=6. Primary myotubes n=4.

We next created a model of NAD ^+^ depletion in muscle cells to assess the consequences for central carbon metabolism. The NAMPT inhibitor FK866 was used to deplete NAD ^+^, and NR as a means to rescue ( Figure 2A). We used 1D- ^1^H-NMR spectroscopy to confirm that the cell models were effective at altering NAD ^+^ levels and quantify key NAD ^+^-related metabolites. Treatment of both fully differentiated C2C12 and primary myotubes with 50 nM FK866 for 48-hours resulted in a 95% reduction in NAD ^+^, which was rescued to above normal levels with 4-hour 0.5 mM NR supplementation ( Figure 2B). NADP ^+^ levels were also significantly supressed by FK866 and restored with NR ( Figure 2C), consistent with previous data ^20^. NAM levels were unchanged with FK866 treatment but increased with the addition of NR ( Figure 2D). Despite basal MeNAM concentrations being higher in primary myotubes compared to C2C12s the levels remained unchanged throughout the different treatment groups ( Figure 2E). Additional metabolites of interest were measured by 1D- ^1^H-NMR ( Table 1). There was no significant change detected in either lactate or alanine production through glycolysis following NAD ^+^ depletion ( Supplementary Figure 2).

**Table 1.** Cellular metabolite concentrations from 1D-NMR spectra. Metabolite concentrations taken from 1D-NMR spectra analysed through Chenomx software.

Metabolite	Cellular Metabolite Concentrations (mM/mg of protein)					
	Control		FK866		FK866 NR	
	Mean	SEM	Mean	SEM	Mean	SEM
1,3-Dimethylurate	0.00	0.03	0.00	0.01	0.00	0.01
1,7-Dimethylxanthine	0.00	0.00	0.00	0.00	0.00	0.05
2-Aminoadipate	0.01	0.01	0.01	0.01	0.01	0.01
2-Aminobutyrate	0.06	0.08	0.06	0.08	0.07	0.07
2-Hydroxyisobutyrate	0.00	0.00	0.00	0.00	0.00	0.01
2-Hydroxyisocaproate	0.02	0.08	0.04	0.06	0.03	0.05
2-Oxoisocaproate	0.00	0.02	0.00	0.01	0.00	0.01
3-Hydroxyisovalerate	0.00	0.00	0.00	0.00	0.00	0.00
3-Methyl-2-oxovalerate	0.01	0.01	0.01	0.01	0.01	0.01
Acetoacetate	0.01	0.01	0.01	0.02	0.01	0.01
Alanine	0.07	0.12	0.08	0.10	0.09	0.15
Arabinose	0.03	0.05	0.04	0.22	0.04	0.04
Asparagine	0.00	0.01	0.00	0.01	0.01	0.01
Choline	0.01	0.02	0.01	0.01	0.01	0.02
Citrate	0.01	0.02	0.01	0.02	0.02	0.02
Dimethylamine	0.00	0.00	0.00	0.01	0.00	0.00
Fumarate	0.00	0.00	0.00	0.00	0.00	0.00
Galactarate	0.01	0.03	0.02	0.03	0.01	0.03
Glucose	0.00	0.01	0.14	0.01	0.01	0.09
Glutathione	0.05	0.06	0.05	0.06	0.06	0.06
Glycine	0.08	0.18	0.11 *	0.21	0.15	0.20
Hydroxyacetone	0.01	0.02	0.01	0.02	0.02	0.02
Imidazole	0.00	0.02	0.01	0.00	0.00	0.01
Nicotinamide N-oxide	0.00	0.00	0.00	0.00	0.00	0.00
O-Acetylcarnitine	0.00	0.01	0.00	0.01	0.00	0.01
O-Acetylcholine	0.01	0.02	0.01	0.02	0.01	0.01
Phenylalanine	0.04	0.12	0.05	0.10	0.03	0.09
Pyruvate	0.01	0.01	0.01	0.02	0.01	0.02
Succinate	0.00	0.01	0.01	0.01	0.01	0.01
Taurine	0.26	0.45	0.29 *	0.47	0.22	0.51
Threonine	0.07	0.09	0.09 *	0.24	0.07	0.09
Tryptophan	0.01	0.02	0.01	0.01	0.01	0.02
Tyrosine	0.05	0.16	0.08	0.14	0.04	0.12
Valine	0.06	0.20	0.10	0.18	0.06	0.16

* p<0.05; Two-way ANOVA performed on all data; Dunnett’s multiple comparison test. n=6.

Although there was no change in overall ATP and ADP levels across the groups in either C2C12 or primary myotubes, the decrease in the PCr/Cr ratio shown in the FK866 samples is evidence the cells are buffering ATP availability in the face of metabolic stress induced by NAD ^+^ depletion ( Figure 2F, G). Also suggestive of altered cytosolic redox is an increase in the lactate/pyruvate ratio, though this was only present in C2C12s ( Figure 2H). To further understand whether the change to the PCr/Cr ratio and redox potential with NAD ^+^ depletion impacted energy metabolism, high-resolution mitochondrial respirometery was conducted and showed lowered maximal respiration following 72-hour FK866 treatment ( Figure 2F).

Together these data show that NAD ^+^ depletion by inhibition of NAMPT alters the energy status of the cell through changes to the PCr/Cr ratio, which can be rescued through short-term NR supplementation, via the NRK enzymes ^30^. The unchanged lactate and alanine data is equally interesting as it highlights how adaptable skeletal muscle cells are at coping with severely depleted NAD ^+^ levels.

### NAD ^+^ depletion causes reversal of glycolytic reactions at NAD ^+^ dependent GAPDH

**Figure 3.**
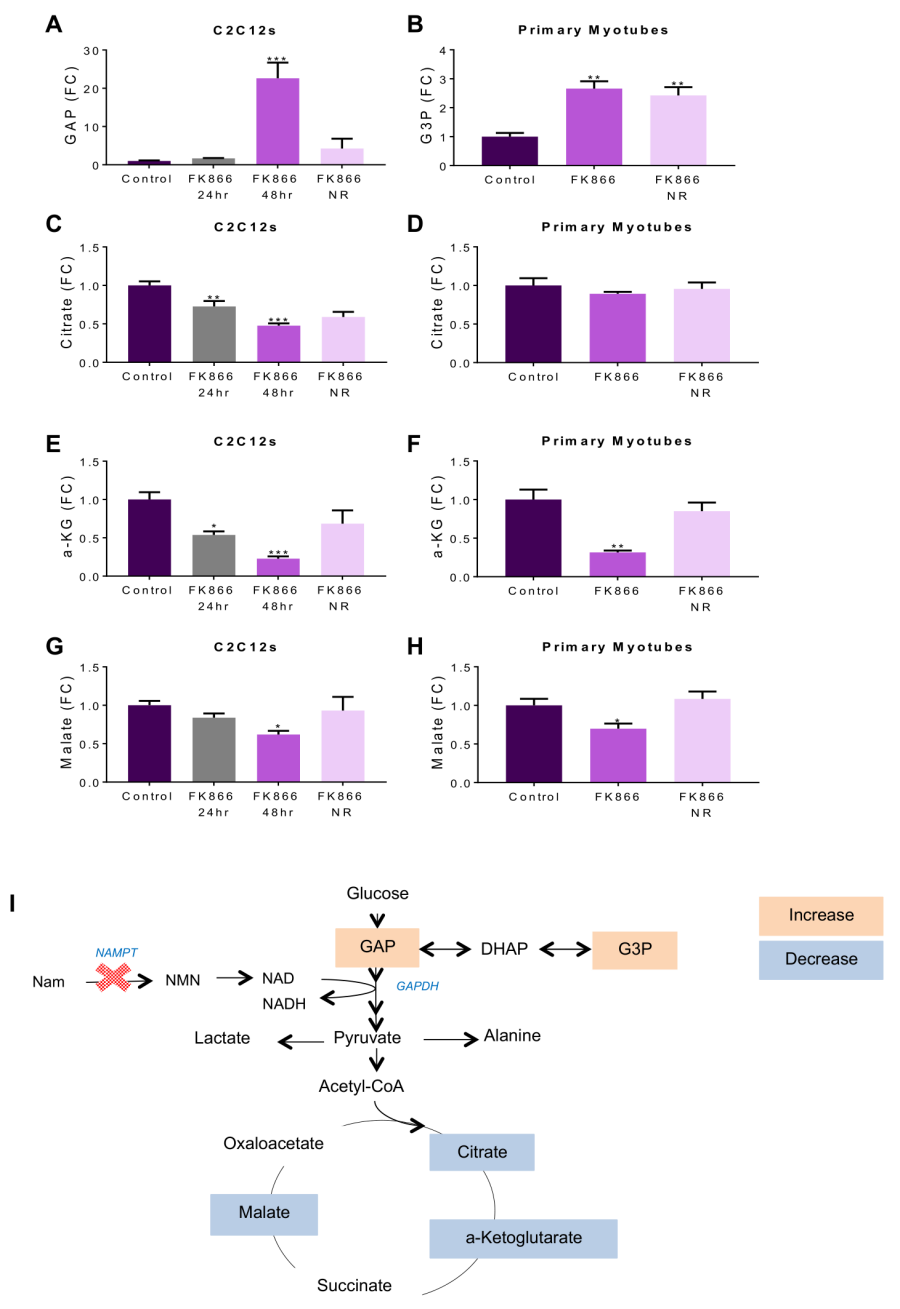
Block in glycolysis at GAPDH following NAD ^+^ depletion. Data shown are fold changes of ion counts from GCMS compared to control in both NAD ^+^ deplete (FK866; 48 h) and replete (FK866; 48 h + NR; 4 h) cells, showing ( **A**) glyceraldehyde-3-phosphate, ( **B**) glycerol-3-phosphate, ( **C**, **D**) citrate, ( **E**, **F**) a-ketoglutarate, ( **G**, **H**) malate. ( **I**) A diagrammatic representation of glycolysis and the TCA cycle in C2C12 and primary myotubes treated with FK866 for 48 h. An increase in ion count compared to control shown in peach and a decrease shown in blue. * p<0.05, ** p<0.01, ***p<0.005; One-way ANOVA performed on raw data; Dunnett’s multiple comparison test, treatment compared to control. All data are the mean ±SEM, n=4.

To understand how changes to energy metabolism pathways within the muscle cell affect metabolite levels, GC-MS ion count analysis was conducted on both C2C12s and primary myotubes. A significant increase in metabolites glyceraldehyde-3-phosphate (G3P) ( Figure 3A) and glycerol-3-phosphate ( Figure 3B), upstream of GAPDH were detected in 48-hour FK866 treated C2C12 and primary myotubes compared to untreated control myotubes. This effect was not observed in the 24-hour FK866-treated cells. Corroborating previous studies, this data is suggestive of an NAD ^+^-dependent reduction in activity of GAPDH, occurring between 24- and 48-hour NAD ^+^ depletion ^19, 20^. NR rescue in FK866-treated C2C12s was able to release the block evidenced by a reduction of GAP to control levels. This was not shown in the primary myotubes, with G3P levels remaining above control following NR supplementation. Presence of a block after 48-hour FK866 treatment was further supported by significantly reduced levels of downstream TCA cycle metabolites citrate, a-ketoglutarate (a-KG), and malate in C2C12s and primary myotubes ( Figure 3C–H). NR rescue was effective at returning all metabolites to control levels in both models. The data is summarised as a schematic in Figure 3I. A number of other metabolites were identified through GC-MS ion count analysis ( Table 2).

**Table 2.** Cellular metabolite ion counts from GCMS. Ion counts taken from GCMS. No significant changes seen between conditions.

Metabolite	Cellular metabolite ion counts							
	Control		FK866 24h		FK866 48 h		FK866 48 h, NR 4h	
	Mean	SEM	Mean	SEM	Mean	SEM	Mean	SEM
Tryptophan	49720	57190	64405	55845	53202	52914	55327	47893
Serine	415367	164737	237489	187173	845253	783737	686931	1055501
Pyruvate	255065	168133	146860	97016	124497	13889	358317	191197
Proline	163717	121858	231244	177360	174065	207804	15691	11678
Malate	147186	112783	114083	89299	73951	64911	142479	159432
Lactate	8345878	7825695	8367333	5174489	7446052	5382583	8998869	3275040
Histidine	646757	551284	741234	488925	706205	654021	605624	362069
Glycine	9116697	7104045	8635419	6465350	7972076	7955743	8503790	6814356
G3P	1174906	826308	1530734	1046045	2273705	2037600	1700887	1223394
Glutamine	942572	1003918	1448528	791723	1545368	1797213	898866	858392
Fumarate	81303	67667	90462	60750	91008	76830	95761	69124
Cysteine	203170	263833	356747	119714	369724	210067	311876	86770
Alanine	1541322	730418	967774	739846	1398345	1367006	1349550	1382444
3-PG	11227	11048	15149	6286	22499	33977	16770	11633

*** p<0.005; Two-way ANOVA performed on all data; Dunnett’s multiple comparison test. n=4.

**Figure 4.**
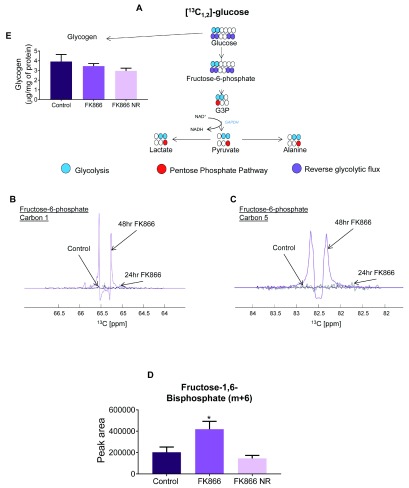
Investigation of reversed glycolytic reactions. Fructose-6-phosphate was used to investigate reversal of glycolytic reactions as it appears before the block at GAPDH. Representative 2D- ^1^H, ^13^C-HSQC spectra were used to show ^13^C label incorporation in both control and FK866. ( **A**) Carbon 1 shows peaks, therefore ^13^C label, in both 48-hour FK866 samples; ( **B**) carbon 5 shows peaks in the FK866 samples, with no presence of ^13^C label in control or 24-hour FK866 samples. ( **C**) Labelling pattern analysis shows how ^13^C _1,2_-glucose is metabolised to 1,2-labelled fructose-6-phosphate during glycolysis, explaining the label incorporation in carbon 1 in both control and FK866 samples. The route to ^13^C label in carbon 5 of fructose-6-phosphate is via 1,2-labelled glyceraldehyde-3-phosphate taking the backward reaction to fructose-1,6-bisphosphate. The presence of label incorporation in carbon 5 of FK866 samples is evidence of reversal of these reactions as a result of an NAD ^+^-dependent build-up of G3P. ( **D**) Glycogen levels of C2C12s. ( **E**) m+6 portion of fructose-1,6-bisphosphate as shown through LCMS analysis. * p<0.05, One-way ANOVA performed on raw data; Dunnett’s multiple comparison test, treatment compared to control. All data are the mean ±SEM, n=3.

We hypothesised that the accumulation of G3P would result in a shift in equilibrium and therefore reversal of glycolytic reactions. In order to study this, we measured fructose-6-phosphate using 2D- ^1^H- ^13^C-HSQC NMR spectroscopy. The resultant labelling patterns from the different metabolic routes are outlined in Figure 4A. We noted elevated ^13^C-label incorporation in carbon 1 of 48-hour FK866 samples compared to both control and 24-hour FK866 samples. This was interesting but could be anticipated as a result of glycolysis using [1,2- ^13^C] D-glucose ( Figure 4B). Investigation of the label incorporation into carbon 5 of fructose-6-phopshate also highlighted 48-hour FK866 samples as showing a differential labelling pattern compared to 24-hour FK866 and control samples ( Figure 4C). We concluded that these labelling patterns could only be produced through reverse glycolytic flux which we suggest to be the result of an NAD ^+^-dependent block at GAPDH. To test this hypothesis using an alternative method we employed LCMS analysis of FK866 samples traced with [U- ^13^C] D-glucose. A significant increase in the m+6 portion of fructose-1,6-bisphosphate is shown with 48-hour FK866 treatment which is normalised with the addition of NR ( Figure 4D). Again, this suggests NAD ^+^ depletion can cause a build-up of metabolites above GAPDH and a reversal of glycolytic reactions. We postulated that this may constitute glycogenesis and therefore result in elevated glycogen levels within the FK866 samples, however glycogen storage appeared to be unaffected ( Figure 4E).

### NAD ^+^ depletion leads to elevated mitochondrial derived aspartate

**Figure 5.**
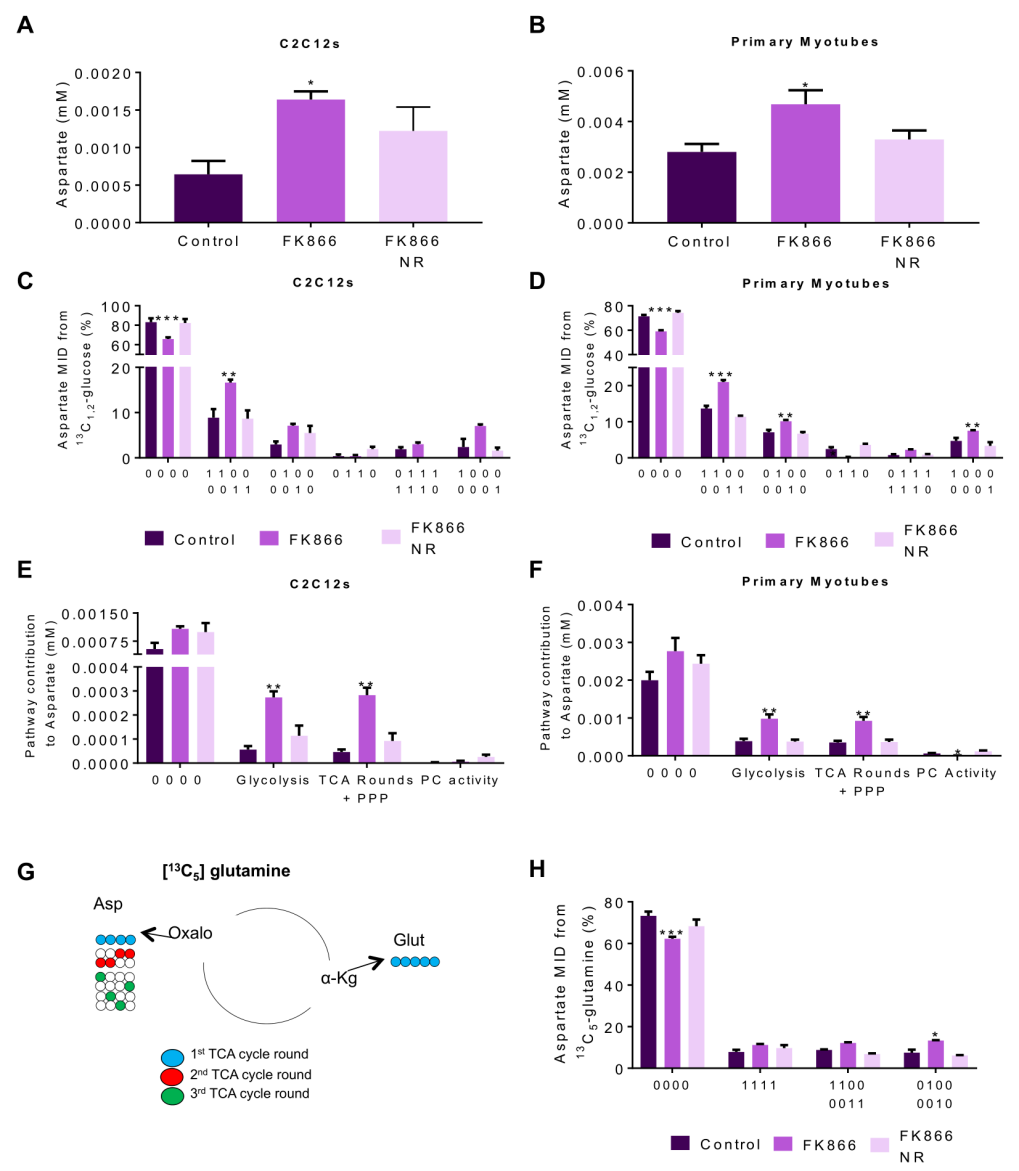
Pathway utilisation for aspartate production is significantly altered with NAD ^+^ depletion. Differentiated C2C12 and primary myotubes were treated with FK866 (50 nM) for 48 hours with and without NR (0.5 mM) for 4 hours. Aspartate concentration in both ( **A**) C2C12s and ( **B**) primary myotubes from 1D-NMR spectra. ( **C**, **D**) Combined analysis of 2D-1H,13C-NMR (13C1,2-glucose) and GC-MS data provides labelling patterns for aspartate; ( **E**, **F**) labelling patterns allow analysis of pathway utilisation for aspartate production. ( **G**) Labelling patterns observed through ^13^C _6_-glutamine tracing. ( **H**) Aspartate MIDs from combined 2D-1H,13C-NMR (13C5-glutamine) and GC-MS. Glycolysis 1100+0011; TCA rounds + PPP 0100+0010, 1111, 0111+1110, 1000+0001; PC Activity 0110. * p<0.05, ** p<0.01, ***p<0.005; One-way ANOVA performed on raw data; Dunnett’s multiple comparison test, treatment compared to control. All data are the mean ±SEM, n=4.

Following the reduced ion count shown of key TCA metabolites, as a result of the GAPDH block in FK866 samples, we were surprised to find a significantly elevated aspartate concentration in both C2C12 and primary myotubes ( Figure 5A, B). Combined analysis of the 2D- ^1^H- ^13^C-HSQC spectra and GC-MS data showed significant alterations to pathway utilisation for aspartate, evidenced by differential labelling patterns occurring after FK866 treatment, which were reversed with an acute dose of NR ( Figure 5C, D). The absolute amount of aspartate produced from each pathway is presented in Figure 5E, F, which not only shows increased aspartate production through glycolysis and the TCA cycle, but also an elevated unlabelled proportion of the metabolite. Generation of unlabelled aspartate is evidence of a non-glucose carbon source being used; in the absence of NAD ^+^ we postulated that the cell may favour glutamine use over NAD ^+^ dependent glycolysis. To investigate this, we used [U- ^13^C] glutamine as a nutrient. Label incorporation into aspartate from glutamine was significantly elevated in FK866 samples, but only in the 2-only labelled and 3-only labelled portion ( Figure 5H). The metabolic route to these labelling patterns is shown in Figure 5G, and depicts increased glutamine use is only evident following multiple TCA cycle rounds. This shows that the cells do not use glutamine as a regular source of carbon for aspartate production under normal conditions, and only begin to do this in response to FK866 treatment.

We postulated that the build-up of aspartate may be a result of impaired malate-aspartate shuttle (MAS) functioning as a result of perturbed cytosolic redox potential, as evidenced by the elevated lactate/pyruvate ratio. We tested this hypothesis using a MAS inhibitor in combination with [1,2- ^13^C] D-glucose. We were successful in increasing the lactate/pyruvate ratio ( Supplementary Figure 3A) and as a result increased NADH oxidation for this reaction in the cytosol. We decreased pyruvate entering the mitochondria, resulting in depleted TCA metabolite levels ( Supplementary Figure 3B, C) and significantly less glucose label incorporation into glutamate ( Supplementary Figure 3F). However, the effect on aspartate did not follow what we had previously seen with NAD ^+^ depletion, with no increased levels and less label incorporation as opposed to more ( Supplementary Figure 3D, E). NR was able to elevate NAD ^+^ levels but had no other effect on the MAS inhibitor treated cells ( Supplementary Figure 3G). Aspartate production following NAD ^+^ depletion may serve as a muscle adaptive response, reversible with NR.

## Discussion

Observation of elevated cellular NAD ^+^ content in response to exercise ^31, 32^ and calorie restriction ^33^ as a metabolic switch signalling augmented energy harvesting in muscle has prompted a focus on ways to increase NAD ^+^ availability for treatment of metabolic disease in humans. Despite measurements linking a decline in NAD ^+^ levels to altered metabolism, there is limited data showing the impact that NAD ^+^ depletion, and also excess, could have on core cellular metabolism in muscle ^10– 12^.

Supplementation of NAD ^+^ precursors in untreated cells results in an ‘NAD ^+^-boost’ above control levels. The methodology employed in this study is key to understanding changes to metabolic pathway usage. It was therefore surprising to discover that there was no alteration to the PPP activity or glycolysis in response to elevated NAD ^+^. It became apparent that skeletal muscle cells rapidly adapt to an ‘NAD ^+^-boost’ by increasing NAD ^+^ clearance through NAM and MeNAM to protect the careful redox balance, seemingly to ensure central carbon metabolism is unaffected ^34^.

A better understanding of the potential consequences of increased NAM and MeNAM clearance following NAD ^+^ excess is important. Increased clearance will lead to increased levels circulating in the blood and ultimately elevated excretion in the urine, with previous studies suggesting high levels may exhibit adverse effects ^35– 37^. With elevated NAM in the blood there is increased substrate availibility for eNAMPT, extracellular NAMPT. The role of eNAMPT, although controversial, has been linked to modulating the immune response and regulating glucose stimulated insulin secretion from β-cells of the pancreas ^38^. This could suggest that excess NAM may affect glucose homeostasis within the body. In addition, these metabolites could also be taken up by various tissues and have shown to cross the blood-brain barrier where there is strong evidence that they exhibit neuroprotection ^39– 41^. However, at high concentrations NAM and MeNAM can have a toxic effect on neurons, as shown in Parkinson’s and Huntington’s disease models ^42– 44^. Similarly, NAM has been shown to be effective for management of hyperphosphatemia in patients with renal disease ^45^; however, it is also suggested to be a uremic toxin contributing to thrombocytopenia ^46, 47^. Whilst there have been studies showing NR safely elevates NAD ^+^ in human blood ^16, 17, 48^ it is important to consider that creating a state of NAD ^+^ excess and increasing clearance of NAM and MeNAM could potentially have unintended effects in the CNS and for kidney function, alongside other possible off-target effects.

Supplementation with NR and NMN in pre-clinical animal studies has shown similar therapeutic effects in disease conditions to preserve metabolic health and ameliorate age-related decline ^15^. This prompted us to utilise both of these supplements to create an NAD ^+^ excess and understand the impact they have on a skeletal muscle cell. These data show a difference between the NAD ^+^ precursors, with a greater elevation of the clearance products, NAM and MeNAM, in NR compared to NMN treated cells. In order for mammalian cells to take up NMN and utilise it for NAD ^+^ synthesis, it must first be converted to NR, which could account for the apparent decreased clearance rate compared to NR ^24, 49^. This is consistent with recent work conducted in mice showing that skeletal muscle metabolises NR faster than NMN ^50^. An alternative explanation for the elevated NAM detected with NR may be due to its relative instability compared to NMN in culture medium, meaning it is possible that we are measuring NAM taken up by cells from the media due to NR degradation as opposed to intracellular NAD ^+^ consumption ^49^.

NAMPT inhibition significantly depletes NAD ^+^, confirming this as the main pathway for basal NAD ^+^ synthesis within skeletal muscle ^12^. NAM levels are carefully balanced between consumption, through NAD ^+^ synthesis, and production, through NAD ^+^ breakdown. NR rescue of FK866 treated cells causes an increase in NAM, suggestive of increased NAD ^+^ breakdown, with NAMPT inhibition preventing NAM being used for NAD ^+^ synthesis, therefore causing an accumulation. However, this build-up did not result in increased clearance through MeNAM possibly due to a delay in establishing NAD ^+^ and NAM excess as FK866 treated cells must recover basal NAD ^+^ levels first.

Evidence of disrupted energy homeostasis was seen with the reduction of the PCr/Cr ratio in NAD ^+^-depleted cells. The hydrolysis of phosphocreatine and the resultant release of a phosphate group provides an alternative route to ATP production, avoiding carbohydrate metabolism and oxidative phosphorylation, which are both NAD ^+^ dependent ^51, 52^. However, the levels of PCr are finite and therefore the cell cannot rely on this method of ATP synthesis indefinitely ^35^. Importantly, supplementation with NR was sufficient to normalise NAD ^+^ levels and prevented requirement for alternative ATP synthesis.

A significant effect of NAD ^+^ depletion is inhibition of GAPDH, the first NAD ^+^-dependent step of glycolysis. The build-up of metabolites before the enzyme, coupled with the decrease in metabolites observed downstream in response to NAMPT inhibition corroborates previous studies in cancer cells and C2C12s ^12, 19^. The advanced analysis conducted through the combined NMR and GCMS methodology allowed us to confirm the reversal of the glycolytic reactions above the block, not just through steady-state metabolite levels, but also through label incorporation into fructose-6-phosphate. This highlighted that although changes to metabolite concentrations were observed after 24-hour FK866 treatment, the inhibition of cytosolic GAPDH, and reversal of glycolytic reactions, is not detected until the 48-hour time-point.

Aspartate is a partner in a number of cellular metabolic energy cycles. Previous work using a malate-aspartate shuttle inhibitor in vascular smooth muscle yielded similar results to our FK866 treatment of an increase in glucose-derived aspartate ^36^. A key limitation of our experiment may be the mechanism of action of the inhibitor, given it indirectly inhibits the shuttle functioning by acting on cytosolic aspartate aminotransferase ^37^. This therefore does not confirm whether a different aspect of the shuttle may be impaired in our model, but does show that the results are unlikely to be a result of compromised cytosolic aspartate aminotransferase activity. Aspartate is also a key component in the purine nucleotide cycle, which is active in skeletal muscle in response to energy depletion, removing AMP to promote ATP synthesis and could provide an alternative use for the aspartate build-up ^53^. Elevated aspartate has also been reported in cells treated with the mitochondrial uncoupler FCCP ^54^, supporting the notion that the effects are a result of perturbed cytosolic redox. Elevated GAP and G3P observed with NAMPT inhibition may represent an attempt to maintain the redox potential within the cytosol, by utilising the glycerol-3-phosphate/dihydroxyacetone phosphate shuttle to regenerate NAD ^+^ and provide protons to the electron transport chain ^55^.

Aspartate could be a marker of a preserved mitochondrial NAD ^+^ pool allowing for continued TCA cycle activity despite cytosolic NAD ^+^ depletion. This is supported by our data indicating mitochondrial respiration still occurs despite severe NAD ^+^ depletion. NAD ^+^/NADH ratios differ greatly between subcellular compartments, with mitochondrial redox more tightly regulated compared to the cytosol ^56, 57^. The notion of a protected mitochondrial NAD ^+^ pool is suggested in other studies, which have indicated that FK866 treatment affects cytosolic NAD ^+^ but does not disrupt the mitochondrial NAD ^+^ pool ^12, 20, 58^. Together this suggests that skeletal muscle is able to resist disruptions to mitochondrial metabolism and maintain oxidative function despite severe NAD ^+^ depletion.

The methods employed in this study provide a high-resolution insight into metabolism and changes to metabolic pathways. Detailed analysis of energy metabolism pathways, as conducted here, could be key to understanding disease pathophysiology and may uncover novel cellular adaptations, which are not seen using other methods.

In conclusion, these data show NR is acutely effective at reversing changes to central carbon metabolism observed following severe NAD ^+^ depletion. NR and NMN in skeletal muscle cells does not impact carbon metabolism but does increase clearance products of NAD ^+^, which may impact other tissues in whole body metabolism. We observed a change in aspartate production with regard to both concentration and route to production. Understanding whether this is a cellular adaptation to low NAD ^+^ or if there is a functional use of the aspartate requires further investigation and could provide insight into how our skeletal muscle copes with energy stress.

## Data availability

Datasets for the study “Metabolic tracing reveals novel adaptations to skeletal muscle cell energy production pathways in response to NAD+ depletion” are available on figshare; DOI: https://doi.org/10.6084/m9.figshare.7271573 ^59^. Included are raw NMR data, GC-MS data, and raw and processed concentrations of metabolites.

Data are available under the terms of the Creative Commons Zero "No rights reserved" data waiver (CC0 1.0 Public domain dedication).

### Extended data

The supplementary figures are available to view on figshare, 

**Supplementary Figure 1. Chronic NR supplementation.**

1D-1H-NMR spectroscopy shows (A) NAD+ and (B) NADP+ concentrations in C2C12s in response to 24-hour NR supplementation. Fold-change compared to control shown for (C) NAM and (D) MeNAM. Energetic status of the cell was investigated through (E) ATP and (F) PCr/Cr ratio. Combined analysis of 2D-1H,13C-NMR (13C1,2-glucose) and GC-MS data of (G) lactate and (H) aspartate. One-way ANOVA performed on raw data; Dunnett’s multiple comparison test, treatment compared to control. All data are the mean ±SEM, C2C12s n=4. DOI: 10.6084/m9.figshare.9785156

**Supplementary Figure 2. Glycolytic outputs, lactate and alanine, unaffected by NAD+ depletion.**

Differentiated C2C12 and primary myotubes were treated with FK866 (50 nM) for 48 hours with and without NR (0.5 mM) for 4 hours. 1D-1H-NMR spectroscopy shows (A) Lactate concentration in C2C12 and primary myotubes (mM/mg of protein). Combined 2D-1H-13C-NMR and GC-MS analysis shows pathway contribution to lactate in (B) C2C12 and (C) primary myotubes. Alanine concentration (mM/mg of protein) in (D) C2C12s and (E) primary myotubes with alanine pathway analysis in (F) C2C12 and (G) primary myotubes. One-way ANOVA performed on all data, with individual pathways assessed independently; Dunnett’s multiple comparison test, treatment compared to control. All data are the mean ±SEM, n=4. DOI: 10.6084/m9.figshare.9785165

**Supplementary Figure 3. MAS Inhibitor does not have same effect as FK866 treatment.**

Differentiated C2C12s were treated with 0.5 mM AOAA (MAS Inhibitor) for 24 hours with or without 0.5 mM NR for 4 hours. (A) Lactate/Pyruvate ratio from GC-MS analysis using the m=2 portion of the MID. GCMS ion count fold changes compared to control for (B) a-KG, (C) succinate and (D) aspartate. MIDs from GCMS analysis for (E) aspartate and (F) glutamate. 1D-1H-NMR spectroscopy shows (G) NAD+ concentration (mM/mg of protein). MAS – Malate aspartate shuttle. * p<0.05, ** p<0.01, ***p<0.005; One-way ANOVA performed on all data, with individual MIDs assessed independently; Dunnett’s multiple comparison test, treatment compared to control. All data are the mean ±SEM, n=3. DOI: 10.6084/m9.figshare.9785168

## Supplementary material

**Supplementary Figure 1. Chronic NR supplementation.**

1D-1H-NMR spectroscopy shows (A) NAD+ and (B) NADP+ concentrations in C2C12s in response to 24-hour NR supplementation. Fold-change compared to control shown for (C) NAM and (D) MeNAM. Energetic status of the cell was investigated through (E) ATP and (F) PCr/Cr ratio. Combined analysis of 2D-1H,13C-NMR (13C1,2-glucose) and GC-MS data of (G) lactate and (H) aspartate. One-way ANOVA performed on raw data; Dunnett’s multiple comparison test, treatment compared to control. All data are the mean ±SEM, C2C12s n=4.

**Supplementary Figure 2. Glycolytic outputs, lactate and alanine, unaffected by NAD+ depletion.**

Differentiated C2C12 and primary myotubes were treated with FK866 (50 nM) for 48 hours with and without NR (0.5 mM) for 4 hours. 1D-1H-NMR spectroscopy shows (A) Lactate concentration in C2C12 and primary myotubes (mM/mg of protein). Combined 2D-1H-13C-NMR and GC-MS analysis shows pathway contribution to lactate in (B) C2C12 and (C) primary myotubes. Alanine concentration (mM/mg of protein) in (D) C2C12s and (E) primary myotubes with alanine pathway analysis in (F) C2C12 and (G) primary myotubes. One-way ANOVA performed on all data, with individual pathways assessed independently; Dunnett’s multiple comparison test, treatment compared to control. All data are the mean ±SEM, n=4.

**Supplementary Figure 3. MAS Inhibitor does not have same effect as FK866 treatment.**

Differentiated C2C12s were treated with 0.5 mM AOAA (MAS Inhibitor) for 24 hours with or without 0.5 mM NR for 4 hours. (A) Lactate/Pyruvate ratio from GC-MS analysis using the m=2 portion of the MID. GCMS ion count fold changes compared to control for (B) a-KG, (C) succinate and (D) aspartate. MIDs from GCMS analysis for (E) aspartate and (F) glutamate. 1D-1H-NMR spectroscopy shows (G) NAD+ concentration (mM/mg of protein). MAS – Malate aspartate shuttle. * p<0.05, ** p<0.01, ***p<0.005; One-way ANOVA performed on all data, with individual MIDs assessed independently; Dunnett’s multiple comparison test, treatment compared to control. All data are the mean ±SEM, n=3.
