# Supplementary figures and images for: Metabolic tracing reveals novel adaptations to skeletal muscle cell energy production pathways in response to NAD + depletion
Source: Wellcome Open Res. 2019 Sep 17;3:147. Originally published 2018 Nov 15. [Version 2] doi: 10.12688/wellcomeopenres.14898.2 (PMC6305244; doi:10.12688/wellcomeopenres.14898.2)

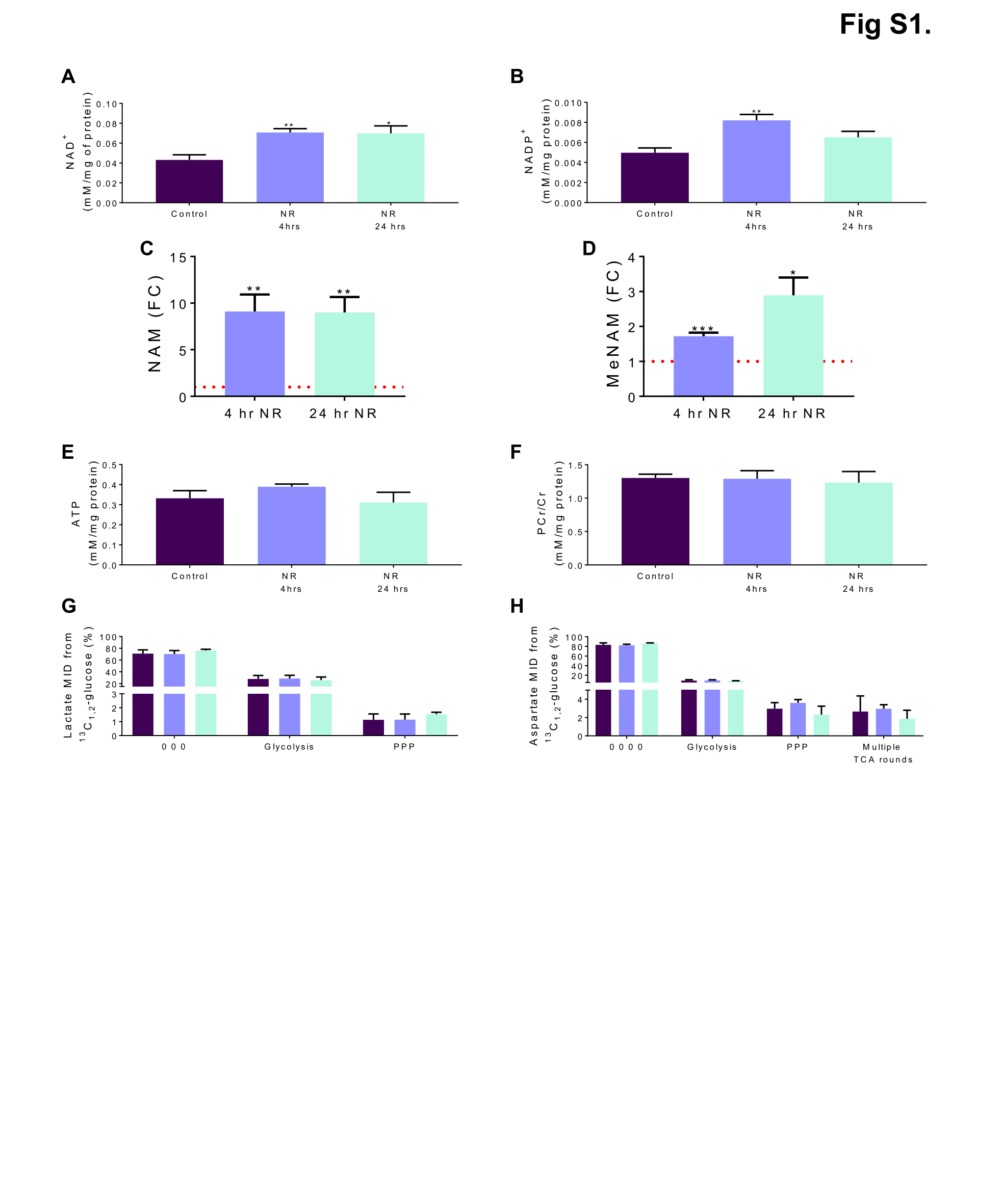

Supplement: Supplementary file 1 [file wellcomeopenres-3-16916-s0000.tgz › a73316ab-52fd-4012-9334-18d5b562bce0_FigS1.tiff]

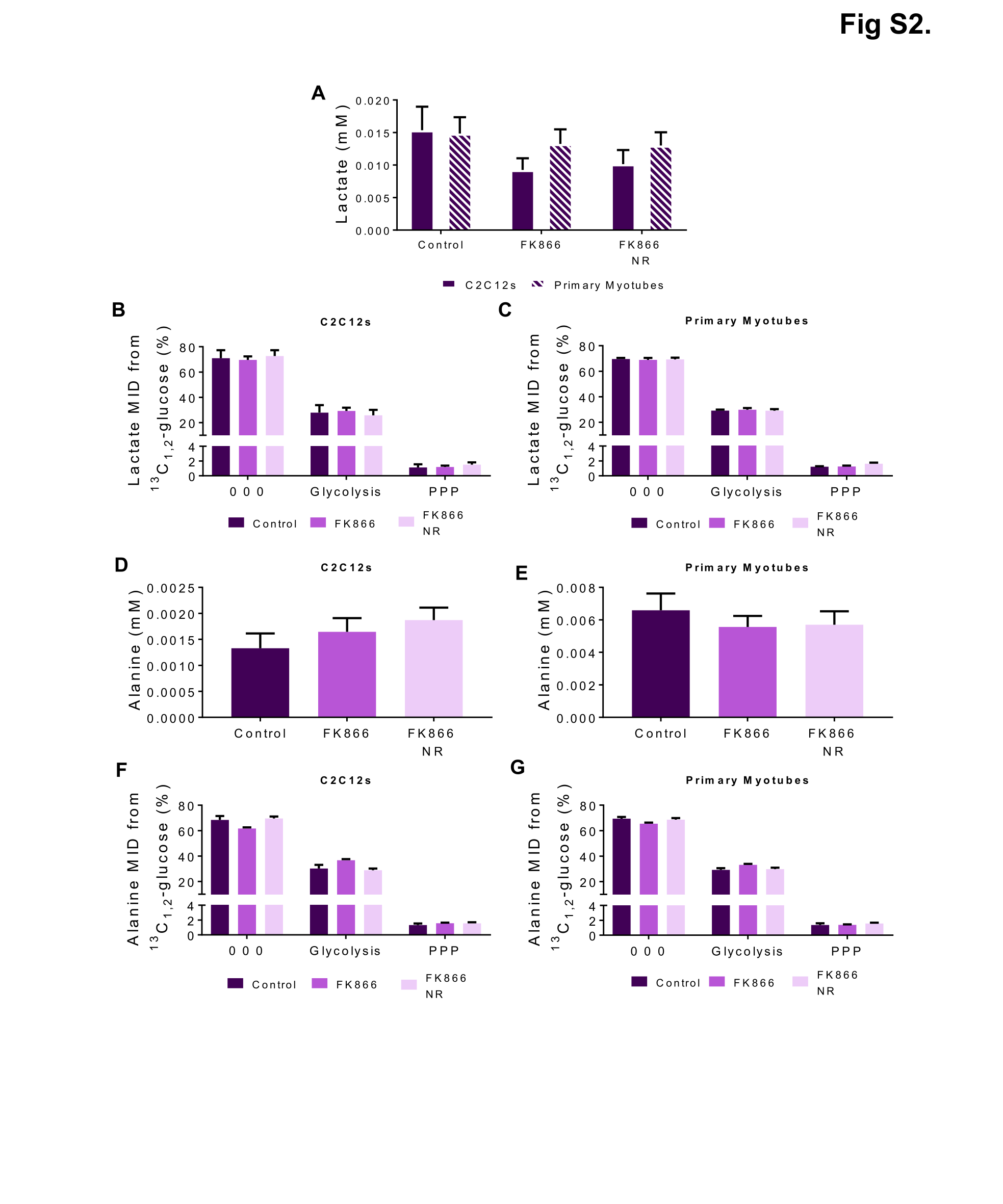

Supplement: Supplementary file 2 [file wellcomeopenres-3-16916-s0001.tgz › 9c12d1b1-dc37-46c2-8b6c-c218aea1dcde_FigS2.tiff]

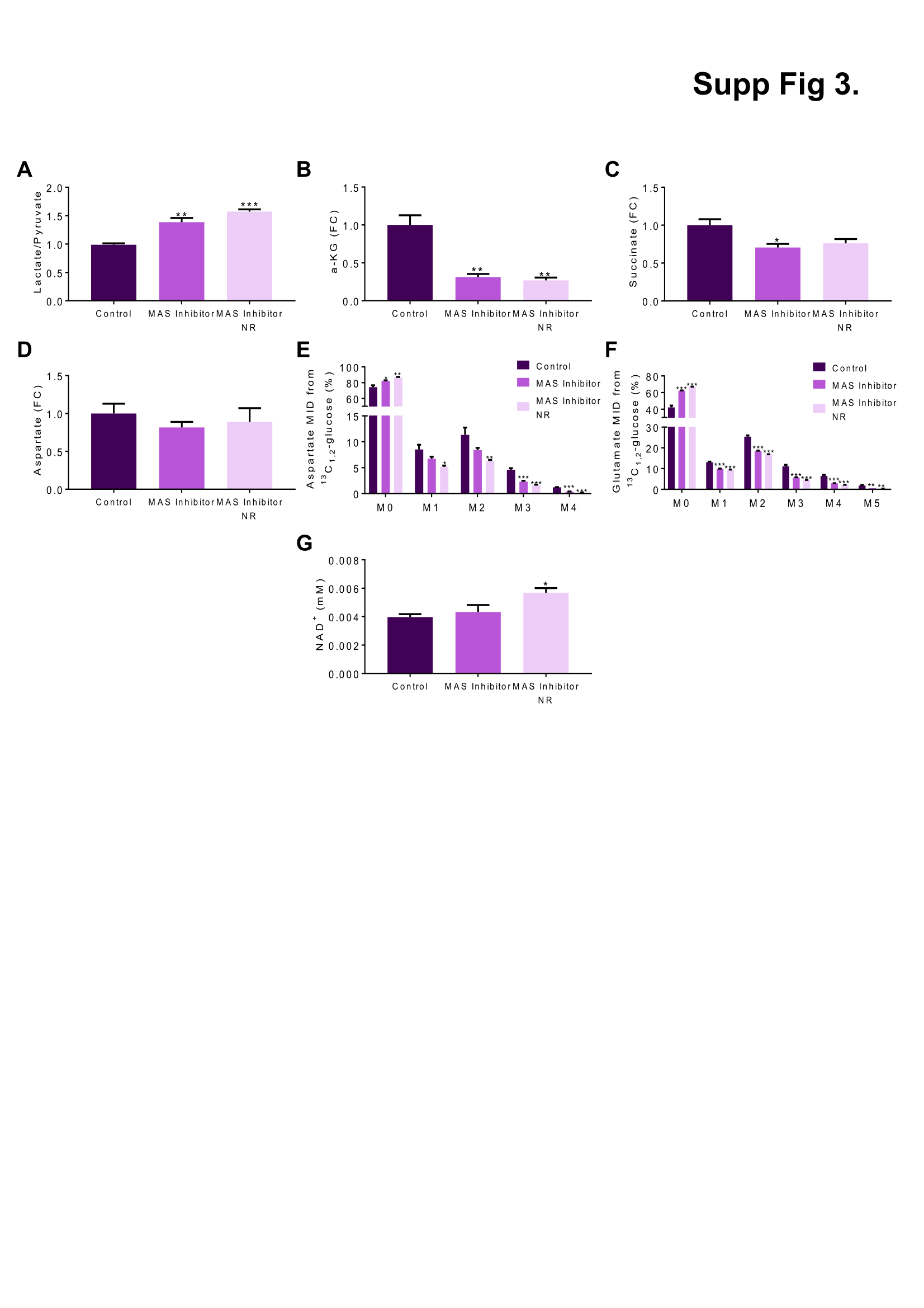

Supplement: Supplementary file 3 [file wellcomeopenres-3-16916-s0002.tgz › 14578bde-1ebe-4880-9830-4adc8f022c54_FigS3.tiff]
