# Supplementary material for: Metabolic tracing reveals novel adaptations to skeletal muscle cell energy production pathways in response to NAD + depletion
Source: Wellcome Open Res. 2019 Sep 17;3:147. Originally published 2018 Nov 15. [Version 2] doi: 10.12688/wellcomeopenres.14898.2 (PMC6305244; doi:10.12688/wellcomeopenres.14898.2)
Supplement: Peer review history

# Peer review: Reviewer response for version 1

**DOI:** 10.21956/wellcomeopenres.16241.r34264

**Published:** 2018-12-12

**Authors:** Joseph A. Baur

**Affiliations:** 1 Institute for Diabetes, Obesity and Metabolism, Perelman School of Medicine, University of Pennsylvania, Philadelphia, PA, USA

Oakey *et al*. apply a combination of NMR and GC-MS based analyses and multiple heavy isotope labeling strategies to provide new insight into the metabolic consequences of perturbing NAD homeostasis in muscle cells. The experiments as presented appear technically sound and the interpretations are reasonable. However, the clarity of the manuscript could be substantially improved be more careful use of language and better explanations for the data presented in many of the figures.

 Specific points:

 1. Flux is inferred or at least implied based on steady-state measurements in some cases. For example, an increased concentration of MeNAM is equated with increased NAM clearance when it could just as easily reflect decreased MeNAM secretion or decreased NAM consumption in another competing pathway. Similarly, lactate is presented as an indication of glycolytic flux. While the authors correctly use lactate labeling pattern as an indication of the relative flux through glycolysis vs. PPP, the distinction should be made that total flux cannot be determined this way. In this regard, monitoring the appearance of lactate in the media would be a very useful addition to the study.

 2. Increased NAM following NR treatment is very likely the result of NR hydrolysis rather than NAD turnover. The authors make this point in the discussion, but at the time when the result is described, the text is suggestive of NAD turnover as the explanation. This seems unlikely given that NMN increases NAD just as much without causing the same degree of NAM accumulation. This could be easily addressed by monitoring the culture media.

 3. There are a number of issues with the presentation of figure 4. Panels D and E appear to be transposed. Panel C is necessary to understand A and B and should probably come first. Since no data are presented relating to the PPP, it could be left out of the diagram for simplicity, and how the diagrammed F-6-P forms relate to the HSQC signals could be more clearly indicated on the figure and in the text. The change in labeling strategy should be indicated on the figure for (m + 6) F-1,6-BP and I am not sure what the label adds here or why the switch from showing F-6-P to showing F-1,6-BP. Can total pools, including labeled and unlabeled be represented for each experiment? 

 4. Figure 5 should have separate diagrams to indicate labeling by glucose or glutamine tracers and the labeling criteria used to determine “glycolysis” or “TCA rounds + PPP” should be indicated more clearly. It appears that the majority of aspartate is unlabeled with either tracer – does this reflect aspartate in the media or another source being used for synthesis?

 5. Wording could be improved in some places

 - in the opening sentence “responsible for driving” implies that NAD levels determine the flux through these pathways, which is not clear above a minimal threshold of NAD concentration

 - “elevate NAD metabolism in human blood” should probably be “elevate NAD concentration”

 - “Detailed analysis of lactate and alanine are key to underpinning changes to glycolysis and the pentose phosphate pathway (PPP) within the cytosol” isn’t quite grammatically correct.

 Minor points:

- The metabolite extraction method is slightly unclear. Are the cell plates on dry ice while they are being washed? How were the cells removed from the plate?
- Figure 1D shows NADP+ is not increased by 4 hours of NR as described in the text, but Figure S1B shows the opposite result
- O2 flux in Figure 2 has no units
- Absolute NAD concentration shown in Figure 2 is much lower than expected
- It would be useful to show NAD depletion at 24 hours of FK866 to help understand why it takes 48 hours to see a major block in GAPDH
- I’m not sure why Figure 3A shows GAP and 3B shows G3P when the rest of the panels show the same metabolite for each cell type. It would be easier to compare if both were shown for both cell types.

I confirm that I have read this submission and believe that I have an appropriate level of expertise to confirm that it is of an acceptable scientific standard.

# Peer review: Reviewer response for version 1

**DOI:** 10.21956/wellcomeopenres.16241.r34265

**Published:** 2018-12-06

**Authors:** Paul Caton

**Affiliations:** 1 Diabetes Research Group, School of Life Course Science, King's College London, London, UK

This is an interesting and clearly written article providing important new information regarding muscle metabolism during NAD excess and depletion. The use of multiple state-of-the-art techniques has uncovered several novel effects of NAD excess and depletion which will be important when considering the utility of NAD boosting compounds as potential therapies for various pathophysiological conditions. 

 Some minor comments/questions:

 1) Details of NMN treatments are missing from the cell treatments section of the methods.

 2) What is the justification for the concentrations of NR and NMN used? How similar are these to physiological levels, either in tissue or serum?

 3) ADPR is mentioned in the schematics showing NAD metabolism. Were the authors able to detect changes in this metabolite in response to any treatments?

 4) Why are some metabolites expressed as fold-change and some as absolute amounts? e.g. in fig1.

 5) Page 8, para 3: ...'via the NRK enzymes'.  Was the role of NRK enzymes assessed here? If not, it might be useful to add a relevant reference there.

 6) Page 13, Discussion Para 3: states NAM and meNAM cross the BBB and are also taken up into the kidney. Are NAM and meNAM taken up into any other tissues? What physiological/pathophysiological effects might this have?

 7) Could increased circulating NAM act as a substrate for eNAMPT (although this function is controversial) and how might that affect the outcomes of NR supplementation in vivo?

I confirm that I have read this submission and believe that I have an appropriate level of expertise to confirm that it is of an acceptable scientific standard.
